# QTL Mapping of Seed Glucosinolate Content Responsible for Environment in *Brassica napus*

**DOI:** 10.3389/fpls.2018.00891

**Published:** 2018-06-27

**Authors:** Yajun He, Ying Fu, Dingxue Hu, Dayong Wei, Wei Qian

**Affiliations:** ^1^College of Agronomy and Biotechnology, Southwest University, Chongqing, China; ^2^Institute of Crop and Nuclear Technology Utilization, Zhejiang Academy of Agricultural Sciences, Hangzhou, China

**Keywords:** *Brassica napus*, quantitative trait loci, seed glucosinolate, environment, flowering time

## Abstract

Glucosinolates (GSLs) are a major class of secondary metabolites. The content of seed GSL is largely regulated by environments in rapeseed (*Brassica napus*). However, the genetic control of seed GSL content responsible for environment in *B. napus* has been poorly understood. In the current study, a doubled haploid (DH) population from a cross between winter and semi-winter lines of rapeseed was grown in two distinct eco-environments, Germany and China, to evaluate the eco-environment effect and dissect the quantitative trait loci (QTL) responsible for environment for seed GSL in rapeseed. The deviation value of GSL content between eco-environments (GSLE) was calculated for each line in the DH population and the QTLs for GSLE were detected. GSLE ranged from −46.90 to 36.13 μmol g^−1^ meal in the DH population, suggesting the prominent eco-environmental effects for seed GSL in rapeseed. Four QTLs for GSLE were identified on chromosomes A04, A06, and A09 explaining 4.70∼9.93% of the phenotypic variation. Comparison of QTLs of seed GSL content between different eco-environments found three QTLs for GSL on A02 from 37.6 to 45.4 cM, A04 from 0 to 17.2 cM, and A09 from 67.0 to 98.6 cM exhibited significant difference of QTL effect between the German and Chinese eco-environments (*P* < 0.01), indicating the environment sensibility of these loci on seed GSL content. Moreover, flowering time (FT), an important environment adaptation trait in plant, was also investigated in this study. Comparative QTL analysis among GSLE, GSL, and FT revealed that three regions on chromosomes A02, A04, and A09 not only exhibited significant differences in QTL effect between Germany and China, but also co-located with the QTL intervals of GSLE and FT. Our results revealed that most of the GSL loci can influence GSL accumulation under different eco-environments, whereas the three QTL intervals on A02, A04, and A09 might be sensitive to the eco-environments for seed GSL content.

## Introduction

Glucosinolates (GSLs) are a major class of secondary metabolites in the Brassicaceae family, such as rapeseed, one of most important oil crops producing edible oil for human diet and protein-rich feed for animals (Wittstock and Halkier, 2000; Bak and Feyereisen, 2001; Leckband et al., 2002; Mikkelsen et al., 2004; Piotrowski et al., 2004; Grubb and Abel, 2006; Halkier and Gershenzon, 2006; Hirai et al., 2007; Francisco et al., 2009; Wanasundara, 2011; Dimov et al., 2012). China with a semi-winter eco-environment and Europe with a winter eco-environment accounts for approximately half of all rapeseed acreages in the world. Since a high content of seed GSLs and their degradation products have anti-nutritional effects on livestock, reducing the seed GSL content has become an important objective of rapeseed breeding (Walker and Booth, 2001).

Quantitative trait loci (QTL) for total seed GSL content have been identified in rapeseed (Uzunova et al., 1995; Howell et al., 2003; Sharpe and Lydiate, 2003; Zhao and Meng, 2003; Basunanda et al., 2007; Hasan et al., 2008; Harper et al., 2012; Javidfar and Cheng, 2013; Li et al., 2014; Fu et al., 2015). Among those results, four QTLs located on A09, C02, C07, and C09 were considered homologs with HAG1 (At5g61420), a key gene controlling aliphatic GSLs biosynthesis in *Arabidopsis* (Uzunova et al., 1995; Howell et al., 2003; Zhao and Meng, 2003; Li et al., 2014). As a major class of secondary metabolites, GSL accumulation was largely regulated by environments (Francisco et al., 2012). Significant environmental effects were observed for GSL accumulation in Brassicaceae (Shelp et al., 1993; Farnham et al., 2004; Yang et al., 2009; Francisco et al., 2012). However, little work has been performed on the deviation of GSL content between eco-environments (GSLE) in *Brassica napus*.

Similar to GSL, flowering time (FT) is also affected by environments, which is an important environment adaptation trait in plant (Johanson et al., 2000; Hall and Willis, 2006; Sherrard and Maherali, 2006; Inouye, 2008; Mendez-Vigo et al., 2011; Fournier-Level et al., 2013; Dittmar et al., 2014). Brassicaceae plants are presented of variation in FT under different growth conditions (Caicedo et al., 2004; Agren and Schemske, 2012). Therefore, comparing the performance of seed GSL and FT between eco-environments is helpful to find the QTLs of seed GSL content responsible for environment in *B. napus*. Although in our previous studies, QTL mapping of seed GSL content and FT was individually performed in the doubled haploid (DH) population (Wei et al., 2014; Fu et al., 2015), the association between FT and seed GSL content was unclear. In addition, a comparison of seed GSL content across eco-environments was lacking; thus the relationship between seed GSL accumulation and growth condition was unknown.

The main objective of this study was to elucidate the genetic basis of seed GSL variation across eco-environments in rapeseed as follows: (1) the deviation of GSLE in *B. napus* was investigated and the QTLs for GSLE were detected. (2) QTLs of seed GSL content between Germany and China eco-environments were compared, and the relationship between seed GSL accumulation and growth condition was investigated. (3) Based on the FT measured by Wei et al. (2014), two more sets of FT values of the DH population were added (grown in Germany in 2012 and in China 2011), the relationship between seed GSL content and FT was determined, and the QTLs among GSLE, GSL, and FT were compared. Our study is of interest in order to better understand the relationship between environment and seed GSL content in rapeseed.

## Materials and Methods

### Plant Materials

A DH population consisting of 261 DH lines of rapeseed, derived from a hybridization between German winter ecotype accession (EXPRESS) and Chinese semi-winter ecotype accession (SWU07), was created in Germany and the seed was introduced to China. The population was investigated for seed GSL content with two replications in two diverse eco-environments, Chongqing and China for 5 years (from 2009 to 2013) which is sub-tropical continental basin of the Yangtze River and, Hohenlieth and Germany for 2 years (between 2008 and 2012) which has the cool maritime climate of the Baltic Sea (Fu et al., 2015).

### Phenotypic Evaluation

Total seed GSL content was measured by near-infrared reflectance spectroscopy with two technical replicates. The deviation value of seed GSL content between eco-environments, abbreviated as “GSLE,” was calculated with the formula GSLE = *M(C)* – *M(G)*, where *M(C)* and *M(G)* represent the mean value of seed GSL content measured in the Chinese and German environments, respectively. Based on the FT reported by Wei et al. (2014), two more sets of FT values of the DH population (grown in Germany in 2012 and in China 2011) were added. The period between sowing and the first flower opening for half of the plants in each plot were defined as FT. The density of plot was followed the local practices.

### QTL Mapping

DNA isolation, development of molecular markers, and construction of genetic linkage groups were described in the previous study (Fu et al., 2015). A total of 316 simple sequence repeat (SSR) markers were finally arranged into 19 *B. napus* chromosomes, spanning a genetic distance of 1,198 cM with an average distance of 3.79 cM between adjacent markers (**Supplementary Data Sheet S1**).

Quantitative trait loci were detected by composite interval mapping procedure using WinQTL cartographer 2.5 software^1^ (Wang et al., 2005). The number of control markers, window size, and walking speed were set to 5, 10, and 1 cM, respectively. A 1,000-permutation test was performed to estimate a significance threshold of the test statistic for a QTL based on a 5% experiment-wise error rate (Churchill and Doerge, 1994).

## Results

### Phenotypic Variation and QTL Mapping of GSLE

To evaluate the eco-environmental effects on seed GSL accumulation, the deviation value of seed GSL content between eco-environments, abbreviated as “GSLE,” was calculated for each line in the DH population (**Supplementary Data Sheet S2**). Continuous segregation was found for GSLE in the DH population. The GSLE exhibited a mean value of 14.02 μmol g^−1^ meal, which ranged from −46.90 to 36.13 μmol g^−1^ meal in the DH population when the eco-environments changed from China to Germany (**Table 1**). The broad deviation of DH lines across eco-environments confirmed the environmental effect on seed GSL content.

**Table 1:** Phenotypic variation of two parental lines and DH population for GSLE.

Traits	EXPRESS	SWU07	DH population		
			Mean ± SD	Range	CV%
GSLE	20.96	15.74	14.02 ± 10.42	−46.90∼36.13	74.35

**FIGURE 1:**
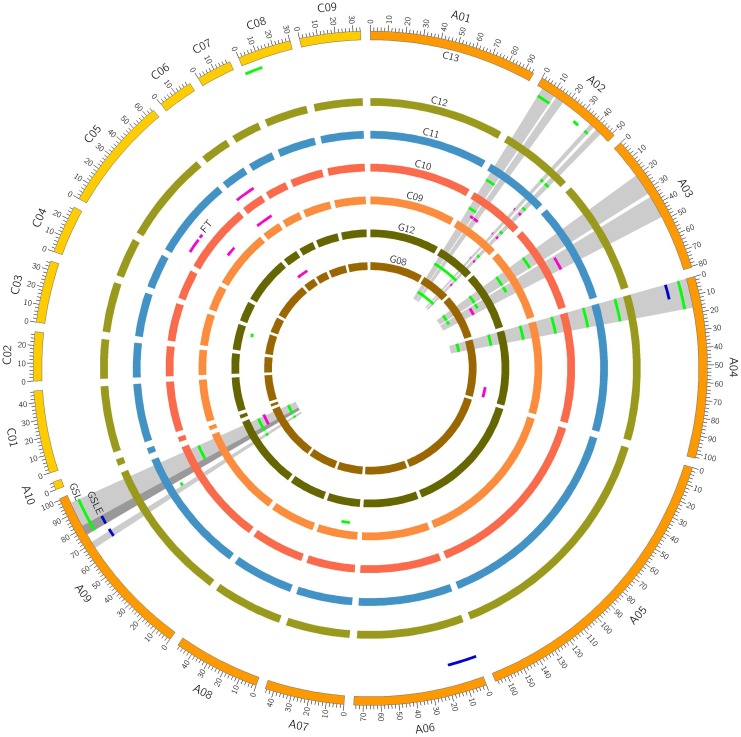
Putative QTLs for seed glucosinolate (GSL) content, flowering time (FT), and the deviation value of seed GSL content between eco-environments (GSLE) on different linkage group.

Quantitative trait loci mapping for GSLE showed that four QTLs were identified distributed on chromosomes A04, A06, and A09 each explained 4.70∼9.93% of the phenotypic variation (**Figure 1** and **Table 2**).

**Table 2:** QTLs associated with seed glucosinolate (GSL) content, flowering time (FT), and phenotypic plasticity of GSL across macroenvironments (GSLE) in DH population of *B. napus.*

QTL	Chr^a^	Pos.^b^ (cM)	LOD^c^	*A*^d^	*R*^2^ (%)^e^	Environments^f^
**Seed GSL content**		
qGSLA02-1	A02	3.2∼11.2	2.53 4.58	−	4.69 5.99	G12, C10, C11, C13
qGSLA02-2	A02	12.6∼17.4	3.62∼5.22	−	3.85∼6.32	G08, G12
qGSLA02-3	A02	29∼32.4	8.68	+	14.74	C13
qGSLA02-4	A02	37.6∼39.9	3.29∼21.57	+	4.02∼31.97	G08, C12, C13
qGSLA02-5	A02	42∼45.4	3.38∼15.96	+	3.35∼20.99	G08, G12, C09, C10, C11, C12
qGSLA03-1	A03	19.9∼32.2	3.75∼7.92	+	6.25∼11.49	G08, G12, C09, C10
qGSLA03-2	A03	34.6∼41.6	2.35∼4.87	+	4.1∼13.5	G08, G12, C09
qGSLA04-1	A04	0∼16.8	5.1∼17.84	+	6.54∼24.87	G08, G12, C09, C10, C11, C12, C13
qGSLA07-1	A07	10.3∼20.2	4.74	−	7.4	C09
qGSLA09-1	A09	67∼70.8	6.69∼8.43	−	7.36∼10.83	G08, G12
qGSLA09-2	A09	71∼73.4	3.19	−	3.77	C11
qGSLA09-3	A09	75.9∼81.4	3.73∼9.57	−	4.58∼12.71	G08, G12, C13
qGSLA09-4	A09	81.4∼96.2	3.75∼8.56	−	4.71∼12.71	G08, G12, C10, C13
qGSLC03-1	C03	22.8∼27.6	3.93	+	4.17	G12
qGSLC08-1	C08	0∼10.4	3.28	+	3.88	C13
**Flowering time**						
qFTA02-1	A02	8.9∼12.2	4.97	+	3.52	C10
qFTA02-2	A02	13.3∼18.3	5.35	+	4.53	C10
qFTA02-3	A02	37.4∼38.2	13.9∼29.98	+	16.78∼35.92	C09, C10, C11
qFTA02-4	A02	43.1∼45.4	14.44∼45.78	+	17.61∼47.68	G12, C09, C10, C11
qFTA03-1	A03	36.2∼47.7	4.06∼6.08	+	4.51∼6.64	G12, C11
qFTA04-1	A04	82.4∼98.8	3.85	+	4.25	G12
qFTA09-1	A09	81.4∼98.5	4.2	−	5.56	G12
qFTC05-1	C05	8.8∼21.6	6.69	+	8.14	C11
qFTC05-2	C05	23.9∼27.1	5.87	+	6.61	C11
qFTC05-3	C05	27.1∼37.3	5.06	+	4.54	C10
qFTC06-1	C06	0∼17.8	3.35∼5.5	−	2.35∼6.56	G12, C10, C11
**Seed glucosinolate GSL content variance responsible in different environments**						
qGSLEA04-1	A04	0.1∼9.1	7.3	−	9.93	−
qGSLEA06-1	A06	0∼18	4.57	−	8.99	−
qGSLEA09-1	A09	67∼71.9	3.49	+	4.7	−
qGSLEA09-2	A09	75.9∼81	5.05	+	6.71	−

*^a^A, C followed by a number designates the linkage group where the QTL was detected. ^b^Length of one LOD score confidence interval. ^c^Peak effect of the QTL (LOD, limit of detection). ^d^Additive effect: positive additivity indicates that the QTL allele originated from the parental line EXPRESS; negative additivity indicates that the QTL allele originated from the parental line SWU07. ^e^Percentage of the phenotypic variance explained by each QTL. ^f^“-G” and “-C” represent trails performed in German and China, respectively and followed by year environment.*

### Comparison of QTLs for GSL Between Different Eco-Environments

Based on our previous study (Fu et al., 2015), a total of 43 QTLs of seed GSL content individually explaining 3.35∼31.97% of the phenotypic variation were identified across eco-environments over years. Of these, 20 QTLs were detected in German eco-environments and 23 QTLs were detected in Chinese eco-environments. In addition, only five QTLs (qGSLA02-3, qGSLA07-1, qGSLA09-2, qGSLC03-1, and qGSLC08-1) were expressed in single eco-environment. The confidence intervals of the remaining 38 QTLs were overlapped between the Chinese and German eco-environments, suggesting that most of the GSL loci can influence GSL accumulation under different growth conditions.

For further screening loci of seed GSL content contributed by the environment, significance test of QTL effect were performed for the QTLs contributed in both eco-environments. Nine overlapped QTLs on chromosome A02 (from 37.6 to 45.4 cM), seven overlapped QTLs on chromosome A04 (from 0 to 17.2 cM), and 10 QTLs on chromosome A09 (from 67.0 to 98.6 cM) exhibited significant difference of QTL effect between the German and Chinese eco-environments (*P* < 0.01) (**Table 2**). The intervals on chromosome A04 with an average QTL effect of 10.55% in the German eco-environment, exhibited notable smaller QTL effect than that in Chinese eco-environment (*P* < 0.01) with an average QTL effect of 23.56%. The intervals on chromosome A09 with QTL effect on average of 4.87% in German eco-environment was much smaller than that in the Chinese eco-environment with an average QTL effect of 9.01% (*P* < 0.01). In contrast, the loci on chromosomes A02 showed an obviously higher QTL effect in the German eco-environment with 20.05 and 6.17% of the QTL effect on average in German and Chinese eco-environments, respectively. These three regions on chromosomes A02, A04, and A09 were possibly involved in the seed GSL content variance responsible for eco-environment variation.

### Comparative Analysis Among GSLE, GSL, and FT

Flowering time is an important environmental adaption trait. Under different growth conditions, Brassicaceae plants are presented of variation in FT. Thus, determining the relationship between seed GSL content and FT will helpful to understand the environmental effects on seed GSL content. In this research, based on our previous study on the GSL measured by Fu et al. (2015) and FT measured by Wei et al. (2014), two more sets of FT values of the DH population were determined. And the 5 years data showed that the winter parental line “EXPRESS” exhibited significantly higher seed GSL content than the semi-winter parental line “SWU07” in both the German and Chinese eco-environments (**Table 3**). In addition, later FT was also observed for “EXPRESS” in both the German and Chinese eco-environments, when compared with “SWU07” (**Table 3**). Comparison of seed GSL content and FT between the Germany and Chinese eco-environments found that the field performance of the DH population grown in Germany (seed GSL content of 29.67 μmol g^−1^ meal and FT of 194.65 days on average), showed significantly lower seed GSL content and later FT than those in China (*P* < 0.01; seed GSL content of 43.21 μmol g^−1^ meal and FT of 183.77 days on average). This suggests the significant environmental contribution to seed GSL accumulation.

**Table 3:** Phenotypic variation of two parental lines and DH population in German and Chinese eco-environments for GSL and FT.

Traits	EXPRESS		SWU07		DH population in China			DH population in Germany		
	China	German	China	German	Mean ± SD	Range	CV%	Mean ± SD	Range	CV%
GSL	40.03	29.02	28.87	16.02	43.21 ± 9.3	22.0∼83.9	21.5	29.67 ± 13.0	8.3∼88.6	43.7
FT	187.1	206.5	166.9	191	183.77 ± 6.4	166∼202	3.5	194.65 ± 4.9	185∼206	2.54

The correlations were analyzed between seed GSL content and FT. The positive and significant correlations were detected between the two traits in both German (*r* = 0.18 in 2012) and Chinese (*r* = 0.33∼0.52 from 2009 to 2011) eco-environments (**Table 4**). This suggests an association between seed GSL content and FT and that later flowering may contribute to the increased seed GSL content. Due to the evolution of FT for environmental adaptation, this significant correlation between seed GSL content and FT reflected an association between seed GSL content and growth condition.

**Table 4:** Correlations among seed glucosinolate (GSL) content and flowering time (FT) in different environments in DH population derived from “EXPRESS” × “SWU07.”

	GSL-G08	GSL-G12	GSL-C09	GSL-C10	GSL-C11	GSL-C12	GSL-C13	FT-C09	FT-C10	FT-C11
GSL-G08										
GSL-G12	0.85^∗∗^									
GSL-C09	0.59^∗∗^	0.59^∗∗^								
GSL-C10	0.57^∗∗^	0.67^∗∗^	0.67^∗∗^							
GSL-C11	0.48^∗∗^	0.55^∗∗^	0.71^∗∗^	0.7^∗∗^						
GSL-C12	0.43^∗∗^	0.52^∗∗^	0.51^∗∗^	0.56^∗∗^	0.62^∗∗^					
GSL-C13	0.55^∗∗^	0.53^∗∗^	0.71^∗∗^	0.59^∗∗^	0.73^∗∗^	0.69^∗∗^				
FT-C09	0.18^∗∗^	0.19^∗∗^	0.43^∗∗^	0.32^∗∗^	0.51^∗∗^	0.38^∗∗^	0.5^∗∗^			
FT-C10	0.15^∗^	0.19^∗∗^	0.44^∗∗^	0.33^∗∗^	0.55^∗∗^	0.37^∗∗^	0.53^∗∗^	0.87^∗∗^		
FT-C11	0.09	0.16^∗^	0.44^∗∗^	0.28^∗∗^	0.52^∗∗^	0.36^∗∗^	0.46^∗∗^	0.69^∗∗^	0.8^∗∗^	
FT-G12	0.15^∗^	0.18^∗∗^	0.32^∗∗^	0.2^∗∗^	0.34^∗∗^	0.23^∗∗^	0.35^∗∗^	0.57^∗∗^	0.62^∗∗^	0.58^∗∗^

*“-G” represents trails performed in German and followed by year environment. “-C” represents trails performed in China and followed by year environment. ^∗^Represents significance at *P* = 0.01 level. ^∗∗^Represents significance at *P* = 0.001 level.*

**FIGURE 2:**
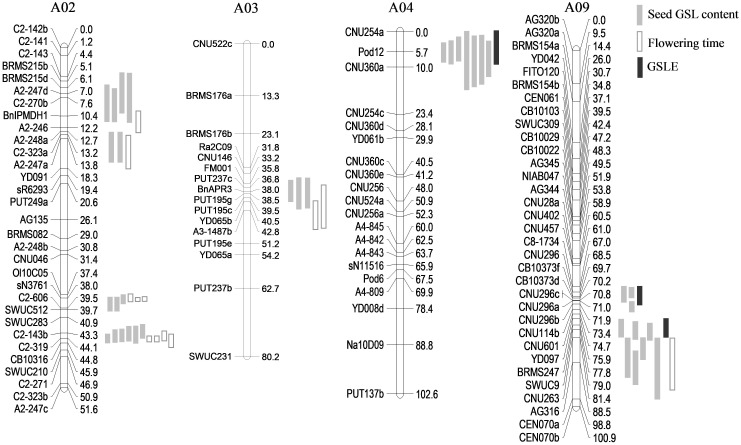
Co-locations of QTLs of seed glucosinolate (GSL) content, flowering time (FT), and the deviation value of seed GSL content between eco-environments (GSLE) on chromosomes A02, A03, A04, and A09.

To facilitate the detection of environment-associated loci of seed GSL content, comparisons of QTLs related to GSLE, seed GSL content, and FT were performed. Eight overlapped GSL QTLs on A02 from 3.2 to 19.5 cM, 12 overlapped GSL QTLs on A02 from 37.4 to 45.4 cM, five overlapped GSL QTLs on A03 from 34.9 to 51.21 cM, and four overlapped GSL QTLs on A09 from 91.4 to 98.6 cM were overlapped with the QTL intervals of FT (**Figure 2** and **Table 2**). Seven overlapped GSL QTLs on A04 from 0 to 17.2 cM and seven overlapped GSL QTLs on A09 from 67.0 to 81.4 cM were overlapped with the QTL intervals of GSLE (**Figure 2** and **Table 2**). These overlapped intervals between GSL and FT/GSLE implied the possible GSL loci associated with environment.

Taken together, based on the QTL comparison between two eco-environments and among three traits, three genetic regions on chromosomes A02 from 37.4 to 45.4 cM, A04 from 0 to 17.2 cM, and A09 from 67.0 to 98.6 cM not only exhibited significant differences in QTL effect for seed GSL between German and Chinese eco-environments, but also overlapped between GSL and FT/GSLE. Thus, we concluded that these three loci could be possibly regarded as candidate loci for seed GSL associated with environment effect.

## Discussion

It is well known that a variety of genetic and environmental factors affect the ultimate metabolite levels in *Brassica* species, although there is still little information about the role that genetics and the environment play on GSL levels. For instance, based on a previous study, GSL level was largely regulated by the daily maximum and minimum temperatures (Francisco et al., 2012). Shelp et al. (1993) observed significant effects of genotype and environment on the GSL content. Farnham et al. (2004) observed significant environmental effects on levels of all GSLs examined. Similarly, in the DH population we detected, it is shown that there are significant variations on GSL accumulation in different environment (Fu et al., 2015). QTL mapping of seed GSL responsible for environment is helpful to better understand the relationship between environment and seed GSL content in rapeseed.

In natural systems, FT is the trait that associates most with determining the accessions suffered from different growing conditions. For instance, the late flowering accessions grown in the Chinese eco-environment were more likely to suffer from high temperature and induce earlier maturation. In this study, the DH population was grown in two highly contrasting eco-environments, China and Germany, to compare the performance of seed GSL and FT between eco-environments. Given that uniform seeding density and field management practices were adopted in both China and Germany, the environmental differences could predominately be attributed to the distinct geographic conditions. Distinct agro-ecologies were exhibited across the two macro-environments, resulting in different climate conditions, especially the lower temperature in Germany. As result, significant lower seed GSL content and later FT were observed in Germany, suggesting lower GSL accumulation and prolonged flowering for rapeseed under cool climate condition. Significant and positive correlations were detected between the traits of GSL and FT in both the German and Chinese eco-environments in this study, suggesting an adaptive association between FT and seed GSL accumulation and that early flowering plants produce higher seed GSL content. Due to FT is an important environment adaptation trait (Hall and Willis, 2006; Mendez-Vigo et al., 2011; Fournier-Level et al., 2013; Dittmar et al., 2014), the significant correlation between seed GSL content and FT reflected that seed GSL content was associated with the growth environment. Meanwhile, GSLE, the deviation value of seed GSL content between eco-environments, was also proposed to assess the environmental variation of seed GSL content. The wide range of GSLE in the DH population across eco-environments validated the environmental effect on seed GSL accumulation.

Though a large number of studies have identified QTLs for seed GSL content in Brassicaceae (Howell et al., 2003; Mahmood et al., 2003; Sharpe and Lydiate, 2003; Zhao and Meng, 2003; Quijada et al., 2006; Basunanda et al., 2007; Hasan et al., 2008; Harper et al., 2012; Javidfar and Cheng, 2013; Li et al., 2014), previous efforts to determine the genetic basis of the low seed GSL trait in *B. napus* were mostly confined to a single eco-environment. This in turn created ambiguity about the roles that heredity and the environment play on seed GSL levels. In our current work, we were interested in exploring of loci, which plant responses to environmental variation. Despite most of GSL QTLs shared genetic control across eco-environments, three regions were found to either exhibited significant differences in QTL effect between German and Chinese eco-environments, or overlapped between GSL and FT/GSLE. Thus these three intervals could possibly be regarded as candidate loci for seed GSL associated with environmental effect. Our study was the first study to elucidate the loci of seed GSL responsible for the macroenvironment and the identified three candidate loci will be useful for the adaptive analysis of seed GSL character.

## Author Contributions

WQ designed the research. YH, YF, and DW performed the research. YH, YF, and DH analyzed the data. YH and YF wrote the paper.

## Conflict of Interest Statement

The authors declare that the research was conducted in the absence of any commercial or financial relationships that could be construed as a potential conflict of interest.

## Footnotes

1 http://statgen.ncsu.edu/qtlcart/WQTLCart.htm

## Supplementary Material

DATA SHEET S1 The raw genotype data.

DATA SHEET S2 The raw phenotype data.
